# Error in Invited Commentary

**DOI:** 10.1001/jamanetworkopen.2024.48060

**Published:** 2024-11-01

In the Invited Commentary titled “Dosing inequities in opioid use disorder treatment trials,”^1^ published October 4, 2024, there was an error in the second paragraph. The sentence starting 4 lines down as “Of note, a larger percentage of White participants who gave consent were screened out prior to randomization (75% vs 64%)” should have been “Of note, a larger percentage of White participants who gave consent were randomized compared with individuals of other races and ethnicities (75% vs 64%).” This article has been corrected.^1^
